# Defects in assembly explain reduced antiviral activity of the G249D polymorphism in human TRIM5α

**DOI:** 10.1371/journal.pone.0212888

**Published:** 2019-03-19

**Authors:** Sevnur Kömürlü, Margret Bradley, Nikolai Smolin, Sabrina Imam, Raymond F. Pauszek, Seth L. Robia, David Millar, Emi E. Nakayama, Tatsuo Shioda, Edward M. Campbell

**Affiliations:** 1 Department of Microbiology and Immunology, Stritch School of Medicine, Loyola University Chicago, Maywood, Illinois, United States of America; 2 Department of Cell and Molecular Physiology, Stritch School of Medicine, Loyola University Chicago, Maywood, Illinois, United States of America; 3 Department of Integrative Structural and Computational Biology, Scripps Research Institute, La Jolla, CA, United States of America; 4 Department of Viral Infections, Research Institute for Microbial Diseases, Osaka University, Suita, Japan; Consejo Superior de Investigaciones Cientificas, SPAIN

## Abstract

TRIM5α is an interferon inducible restriction factor which contributes to intrinsic defense against HIV infection by targeting the HIV capsid protein CA. Although human TRIM5α (huTRIM5α) does not potently inhibit HIV-1 infection, the ability of huTRIM5α to exhibit some control of HIV-1 infection is evidenced by a single nucleotide polymorphism in huTRIM5α which substitutes aspartic acid to glycine at position 249 (G249D) in the L2 region and is associated with higher susceptibility to HIV-1 infection. To understand the mechanistic basis for the reduced antiviral activity, we employed biophysical and cell biological methods coupled with molecular dynamics simulations to compare WT and the G249D polymorphism of huTRIM5α. We investigated the differences in conformational dynamics of rhesus and huTRIM5α Coiled Coil–Linker 2 (CC-L2) dimers utilizing circular dichroism and single molecule-Fluorescence Energy Transfer (sm-FRET). These methods revealed that the G249D dimer exhibits secondary structure and conformational dynamics similar to WT huTRIM5α. Homology modelling revealed that G249 was present on the hairpin of the antiparallel dimer, in a position which may act to stabilize the adjacent BBox2 domain which mediates the inter-dimeric contacts required for the formation of TRIM5 assemblies. We therefore asked if the G249D mutant forms assemblies in cells with the same efficiency as WT protein by expressing these proteins as YFP fusions and quantifying the number of assemblies in cells. In cells expressing comparable amounts of protein, the G249D mutant formed fewer assemblies than WT protein, in agreement with our homology modeling predictions and molecular dynamics simulations of dimers and higher oligomers of TRIM5α, providing a mechanistic explanation of the reduced antiviral activity of the G249D polymorphism.

## Introduction

TRIM5α is a potent restriction factor against retroviruses that mediates a post-entry block to infection by faciltating abortive disassembly of capsid core [1–3]. Like all other TRIpartite Motif (TRIM) family members, TRIM5 alpha contains an N-terminal RING, BBox2 and coiled-coil (CC) domains which define the TRIM family [4, 5].The C-terminal of TRIM5α contains a SPRY/PRY domain connected to the RBCC motif by a linker region (L2). The RING domain mediates E3 ubiquitin ligase activity upon higher order assembly [3, 6–8], the BBox2 domain mediates the higher-order hexagonal assemblies of individual TRIM dimers comprised of the CC domain and L2 region [9–17]. The lattice spacing of TRIM5α hexagonal assemblies matches with the HIV capsid, allowing formation of these assemblies around the viral capsid [18–20]. It is also known that formation of TRIM5α assemblies around the HIV capsid results in the abortive disassembly of the capsid core through a poorly defined mechanism.

TRIM5α differ from other TRIM family members by its C-terminal SPRY/PRY domain which binds to the HIV capsid and has species specific variability [21–24]. As a result of an intensive selective pressure, SPRY/PRY domains of different primates have evolved to generate species specific restriction potencies against different retroviruses [22, 23, 25]. For example, while rhesus TRIM5α (rhTRIM5α) provides potent restriction against HIV-1 and N-tropic murine leukemia virus (N-MLV), human TRIM5α (huTRIM5α) does not potently inhibit HIV-1 infection but does provide strong restriction against N-MLV [1, 21], and a single nucleotide change in SPRY domain restores potent restriction of HIV-1 by huTRIM5α [23, 24]. Despite the fact that huTRIM5α does not prevent HIV-1 infection or progression of HIV-1 infected patients to AIDS, numerous studies have observed measurable antiviral activity against HIV-1 [1, 26–29], and one recent study suggests that huTRIM5α can potently inhibit HIV-1 infection in Langerhans cells [30]. It was also shown that stabilized huTRIM5α exhibited potent HIV-1 inhibition of HIV-1 replication *in vivo* in human T cells transferred to immunocompromised mice [27]. The clinical relevance of huTRIM5α was shown by studies on escape mutations from cytotoxic T lymphocyte which increases sensitivity of the HIV-1 to huTRIM5α [31, 32], and a recent study identified TRIM5α dependent innate activation pathways likely to contribute HIV-1 resistance by elite controllers [33]. Moreover, the ability of huTRIM5α to inhibit HIV-1 infection in humans has also been supported by studies of huTRIM5α single nucleotide polymorphisms (SNPs), which reveal some polymorphisms are associated with increased susceptibility to HIV-1 infection and disease progression [34–38]. One of these SNPs is located in L2 region which substitute aspartic acid to glycine at position 249 (G249D) and is associated with higher susceptibility to HIV-1 infection and higher viral titers in HIV infected patients [39–41].

In this study, we sought to determine the mechanistic basis for reduced antiviral activity of G249D mutant compared to wild type (WT) huTRIM5α. Our previous studies have shown that restriction of HIV-1 by rhTRIM5α was affected by α-helical content of CCL2 dimers and dynamic conformational changes in the L2 region [10, 14, 42]. To test whether huTRIM5α exhibited similar behavior, and to determine if the G249D substitution impacted these characteristics, we employed biophysical methods including circular dichroism and single molecule-Fluorescence Energy Transfer (sm-FRET) and compared WT and the G249D polymorphism of huTRIM5α. These studies did not reveal any significant differences between WT and G249D huTRIM5α.

It is also known that TRIM5α forms cytoplasmic accumulation of proteins in cells called cytoplasmic bodies, like other TRIM family members forming protein bodies in cytoplasm or nucleus [1, 26, 43]. It has been suggested that cytoplasmic body formation is a manifestation of higher order assemblies observed *in vitro* [15, 18] which was supported by the live cell imaging experiments [44]. Our homology modelling for WT and G249D huTRIM5α indicated that the formation of the higher order assemblies might be affected by G249D substitution due to its location in hairpin structure in close vicinity to the BBox2 domain. For this reason, we investigated formation of cytoplasmic bodies by fluorescence imaging of cells expressing WT and the G249D huTRIM5α and observed that cytoplasmic body formation is reduced by the G249D mutant. We attributed the decreased antiviral activity of G249D huTRIM5α to deficiency in formation of higher order assemblies which is supported by molecular dynamic simulations. Our work reveals a critical residue in L2 region of huTRIM5α affecting formation of high order assemblies indirectly by altering positioning of the BBox2 domain.

## Materials and methods

### Recombinant DNA

6xHis tagged CCL2 dimers were cloned into pET-15b vector by using NdeI and BamHI restriction sites. The cysteine residues for labelling were introduced by primers containing the codon for cysteine against C-terminal end of CCL2 peptides. The G-to-D substitution was introduced by site-directed mutagenesis.

### Protein expression and purification

E. Coli BL21 (DE3) cells transformed to express recombinant 6xHis tagged CCL2 peptides were grown in 250 ml Luria broth containing 100 μg/ml carbenicillin (Invitrogen) until optical density reached to 0.6 at 600 nm. The protein expression was induced by addition of 1mM isopropyl β-D-1-thiogalactopyroniside (IPTG, Invitrogen) and shaking cultures for 4 hours at 37 ^0^C. Cell pellets were resuspended in lysis buffer containing 50 mM Na_2_HPO_4_(pH 8), 8M Urea, 500 mM NaCl, 10mM imidazole, 1% Triton X-100, 0.5 mg/ml lysozyme (Sigma) and a protease inhibitor cocktail (Roche) and lysed by sonication (6x10s on ice 40% output). Cell lysates were clarified by ultracentrifugation at 10000 rpm (Beckman Ti 41 rotor) for 30 min at 4 ^0^C. The supernatants were incubated with Talon affinity resin (Clontech) at 4°C for 1 hour while rotating to facilitate binding. The resin was placed in a disposable gravity column (Clontech) and washed with buffer containing 50 mM Na_2_HPO_4_ (pH 8), 8 M Urea, and 500 mM NaCl. The 6xhis tagged peptides were eluted by buffer containing 50 mM Na_2_HPO_4_ (pH 8), 8 M Urea, 500 mM NaCl and 300 mM imidazole. The eluents were dialyzed at 4°C by using 10000MW Slid-A-Lyzer Dialysis Cassettes (Thermo) in decreasing concentration of urea (4 M, 2 M and 0 M). To remove aggregates, the peptides were spun at 13000 rpm and the concentrations were determined by absorption at 280 nm. The purity of the protein solutions were assessed Coomassie staining of SDS-PAGE gels. The α-helical content of the peptides were determined by using circular dichroism data and CDPro software (CD data were collected aby Protein-Peptide Metabolic Lab at University of Illinois at Chicago). Spectra were collected in 0.1 cm path-length cuvette for 4 independent batches of purified peptides. The helical contents were determined by using Selcon II software.

### Protein labelling

To disrupt cysteine bridges, purified CCL2 peptides were incubated with 10 fold excess TCEP overnight at 4°C. The donor (Alexa 488 C5-maleimide, Life technologies) and acceptor (Alexa 594 C5-maleimide, Life technologies) dyes dissolved in DMSO were added in three-fold excess and incubated overnight at 4°C. The labelling reaction was halted by addition of 1μl of βME. The reaction mixture is dialyzed at 4°C by using 10000MW Slid-A-Lyzer Dialysis Cassettes (Thermo). Labelled proteins subjected to SDS-PAGE gels and imaged by GE Typhoon gel Trio+ Gel scanner using 526 nm and 610 nm filters for each fluorophore and pseudo-colored by Image Quant TL.

### Single molecule FRET measurements

The labelled CCL2 peptides were immobilized on glass coverslip surfaces for sm-FRET measurements. Glass coverslips and slides were cleaned and passivated with maleimide-PEG and 5% biotin-PEG as previously described [42]. A microfluidic chamber was formed by passivated glass cover slips and slides and incubated with neutrAvidin (0.2 mg/ml) for 5 minutes and washed with imaging buffer (50 mM HEPES, 150 mM NaCl, 0.1 mg/BSA and 2 mM Trolox used as an oxygen scavenger) which is followed by incubation with 10 nM biotin conjugated penta-his antibody (Qiagen). After washing with the imaging buffer, the chamber is incubated with the his-tagged labelled peptides (2 nM) for 20 minutes and washed again with the imaging buffer to remove access peptides.

Sm-FRET data were acquired on a through objective total internal reflection fluorescence microscope (Nikon Ti-e) using a 488 nm Argon laser (Spectra Physics) as an excitation source. Fluorescence emission was collected by an inverted oil-immersion objective (100x) with 1.49 NA, split into donor and acceptor channels using a Optosplit II Image Splitter (Cairn Research) with a custom ordered splitter filter cube (ET 535/70, ET 645/75, T600lpxr-UF2 = 400, UF1 = 300) and imaged on an cooled charged coupled camera (Andor iXon 887) with an integration time of 100 msec/frame. Movies were recorded with a custom data acquisition package and individual donor/acceptor intensity traces were extracted using IDL scripts (Ha lab, John Hopkins School of Medicine). Sm-FRET data were analyzed as described previously [42].

### Cell culture reagents and methods

Expression plasmids for YFP or HA tagged huTRIM5α were described previously [10, 26]. Site directed mutagenesis was used to generate G249D mutants. CRFK and HEK293T cells were cultured in complete Dulbecco’s modified Eagle’s medium (DMEM) containing 10% fetal bovine serum, penicillin (100 U/ml), streptomycin (100 μl/ml) and ciprofloxacin (10 μl/ml). HEK293T cells were transfected with expression plasmids along with envelope like glycoprotein from vesicular stomatitis virus (VSV-G) and retroviral packaging plasmid (pCig-B) and the vectors were used for transduction. Forty-eight hours after transduction, CRFK cells were selected in G418 (400 ug/ml). Stable cell lines expressing YFP tagged huTRIM5α were subsequently analyzed by imaging and immunoblotting. Fluorescence intensities collected by FACSCanto II flow cytometer (Becton, Dickinson) and geometric means are used for mean fluorescence intensity (MFI) calculations of 5 biological replicates to compare expression levels. For immunoblotting, whole-cell lysates were harvested and lysed by lysis buffer (100mMTris [pH 8.0], 1%NP-40, 150 mM NaCl) containing a protease inhibitor cocktail (Roche Applied Science, Indianapolis, IN, USA). The protein contents were quantified by BCA protein assay kit (Thermo Fischer) and equal amounts of protein boiled with Laemmli 2X SDS sample buffer for 5 min. They were loaded onto a 12% polyacrylamide gel for SDS-PAGE and separated by electrophoresis. After separation, proteins were transferred onto nitrocellulose membranes and detected by incubation with the following antibodies: anti-HA (clone 3F10) conjugated to horseradish peroxidase (HRP) (Roche Applied Science, Indianapolis, IN, USA), or anti-green fluorescent protein (GFP) (Clontech Laboratories, Inc., Mountain View, CA, USA), followed by secondary antibody conjugated to HRP (Thermo Fisher Scientific,Waltham, MA, USA). SuperSignal West Femto chemiluminescent substrate (Thermo Fisher Scientific, Waltham, MA, USA) were used and chemiluminescence was detected by using a UVP EC3 imaging system (UVP LLC, Upland, CA, USA).

### Cytoplasmic body assembly assays

CRFK cells stably expressing equivalent amounts of YFP tagged (or HA tagged) human WT or G249D TRIM5α proteins were adhered on to fibronectin-treated coverslips. They were subsequently fixed with 3.7% formaldehyde (Polysciences) in 0.1M PIPES (piperazine-N,N^`^-bis(2-ethanesulfonic acid)] (pH 6.8). Nuclei were stained with DAPI. Images were collected with a DeltaVision microscope (Applied Precision) detected with a digital camera (CoolSNAP HQ:Photometrics) using a 1.4 numerical aperture lens. They were deconvolved with SoftWoRx deconvolution software (Applied Precision). Z stack images of each cell line were collected using identical acquisition parameters. Deconvolved images were analyzed by using the Surface Finder function of Imaris software (Bitplane). Surfaces were created around YFP huTRIM5α proteins and maximum fluorescence intensities on each surface were quantified. Background fluorescence intensities were calculated and used to set thresholds. By using Imaris software the number of puncta was quantified for 168 cells for three biological replicates.

### Restriction assays

HEK293T cells were transfected to produce vesicular stomatitis virus G protein (VSV-G)-pseudotyped R7ΔEnvHIV-GFP as previously described [44]. For production of VSV-G pseudotyped N-MLV, transfection was done with equal amounts of VSV-G, pCigN and GFP-MLV reporter virus. Equivalent number of CRFK cells were plated in 24-well plates and infected with serial dilutions of virus. They were subjected to spinoculation for 2 hours (12000x g at 13°C), then the medium was changed. Cells harvested 48 hours post infection and fixed in a 1% formaldehyde solution. Viral infectivity was assessed by determining relative number of green fluorescent protein expressing cells using a FACSCanto II flow cytometer (Becton, Dickinson).

### Molecular dynamics simulation

MD simulations were performed using GROMACS [45, 46] with the CHARMM 36 force field [47] and TIP3P water model [48]. We used recently published crystal structures of rhesus Bcc miniTRIM oligomers [15] to create starting structures for trimers and monomers of rhesus Bcc miniTRIM. To create human WT and G249D Bcc miniTRIM we used Modeller software package [49]. The starting systems were minimized using the steepest descent method for 1000 steps, and then were solvated in a rectangular water box with a minimum of 20 Å from the surface of the protein to the edge of the solvent box. Na^+^ and Cl^-^ ions were added to the solution to neutralize the charge of the system and to produce an ion concentration of 150 mM. The Particle Mesh Ewald (PME) method [50, 51] was used to describe long-range electrostatic interactions. Molecular dynamics simulations were carried out with an integration time step of 2 fs. To reach the target temperature (300 K) and pressure (1 bar), the Berendsen method [52] was used with relaxation time of 0.1 ps. After a 100 ps equilibration, production simulations were performed in the NPT ensemble using the Nose-Hoover thermostat [53, 54] and a Parrinello-Rahman barostat [55, 56] with relaxation times of 1.0 ps. Three independent production runs for each system were carried out for 100ns each.

## Results

### Restriction is reduced by G249D polymorphism in human TRIM5α

**Fig 1:**
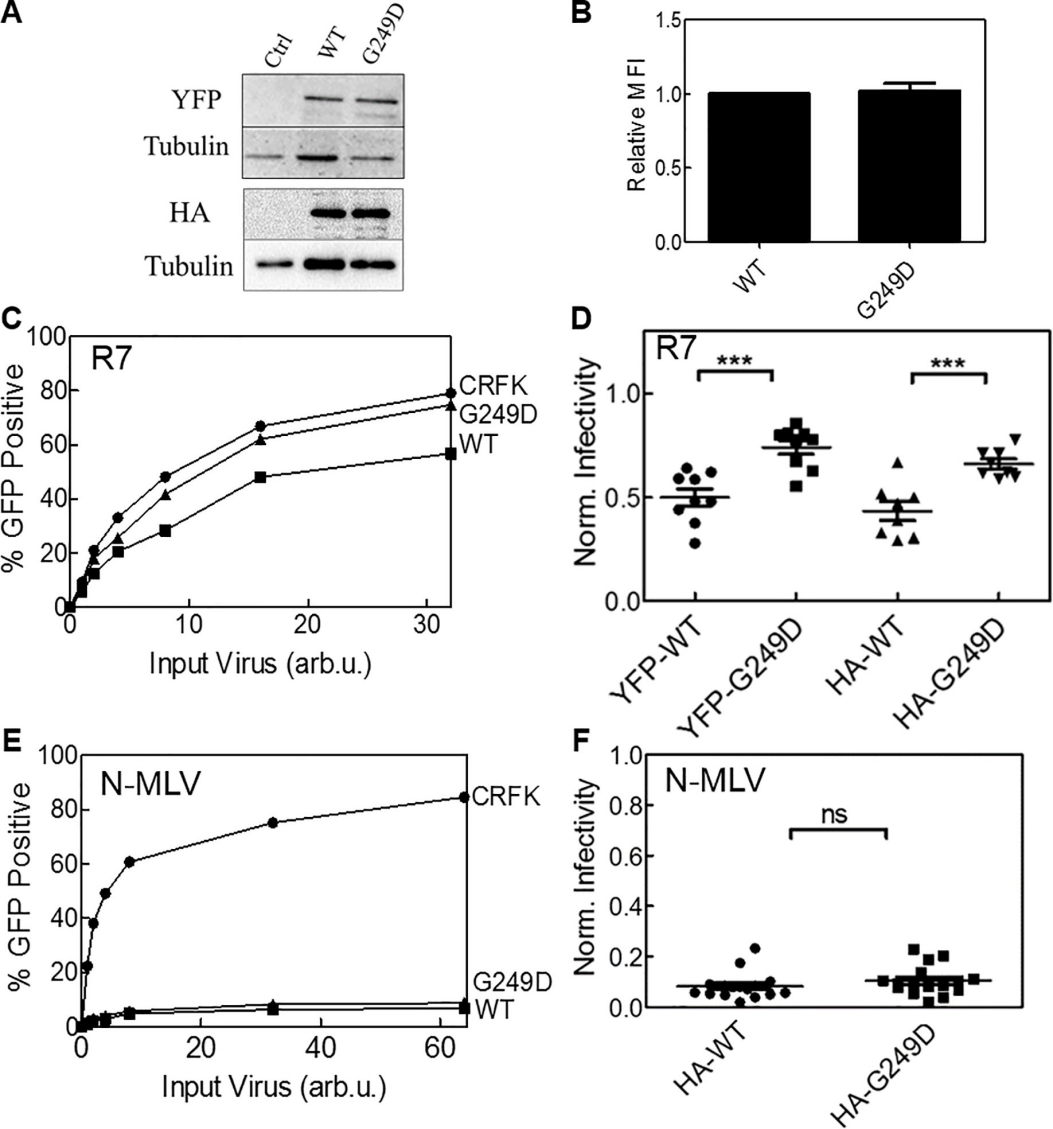
The G249D polymorphism of huTRIM5α exhibits reduced restriction of HIV-1 but not N-MLV. CRFK cells transduced to stably express YFP and HA tagged human TRIM5α. (A) Protein expression of YFP and HA tagged human WT, G249D TRIM5α and untransduced (control) cell lines by immunoblot. Tubulin used as a loading control. (B) Relative mean fluorescence intensities of WT and G249D TRIM5α by flow cytometry. Error bars represent SEM of 5 biological replicates. (C) Equivalent number of CRFK cells infected by R7ΔEnvGFP or N-MLV (E) by a serial of dilutions. Percent GFP positive cells quantified by flow cytometry 48 hours post infection. (D and F) Normalized infectivity for cell lines expressing YFP and HA tagged human WT, G249D TRIM5α. Normalization done by using percent GFP positive cells of untransduced cell line for virus concentrations resulting less than 35% infectivity (p<0.05 compared to the WT).

We transduced the feline CRFK cell line, which does not express endogenous TRIM5α [57], with a vector expressing YFP fusions of WT and G249D huTRIM5α. Expression of both proteins was found to be equivalent in CRFK cells by western blot and MFI (mean fluorescence intensity) (Fig 1A and 1B). The restriction efficiency by WT and G249D huTRIM5α were compared by infecting equivalent number of CRFK cells with HIV-1 GFP reporter virus pseudotyped with VSV-G envelope. The percent GFP positive cells were obtained by flow cytometry (Fig 1C). We observed modest but significant restriction of HIV-1 infection in cells expressing WT huTRIM5α, similar to previous reports [1, 26]. This restriction was reduced in cells expressing G249D huTRIM5α, in agreement with our previously published studies [40]. We quantified the change in infectivity of WT or G249D huTRIM5α expressing cell lines by normalizing percent GFP positive cells to the untransduced cell line infected with the same viral titer. Infectivity levels lower than 35% were used for our normalization calculations (Fig 1D). Similar results were observed in CRFK cells expressing Hemagglutinin (HA) tagged WT or G249D huTRIM5α (Fig 1D).

As our restriction assays showed reduced restriction of HIV-1 by G249D polymorphism, we also asked if the restriction of N-MLV was relieved by this polymorphism. We assessed the N-MLV restriction in CRFK cells stably expressing HA tagged human TRIM5α (Fig 1E). Notably, we observed very similar restriction of pseudotyped N-MLV by our WT and G249D expressing cells lines. This data demonstrates that perturbation caused by G249D substitution does not affect the restriction of N-MLV.

### G249D polymorphism in human TRIM 5α does not affect α-helical content

**Fig 2:**
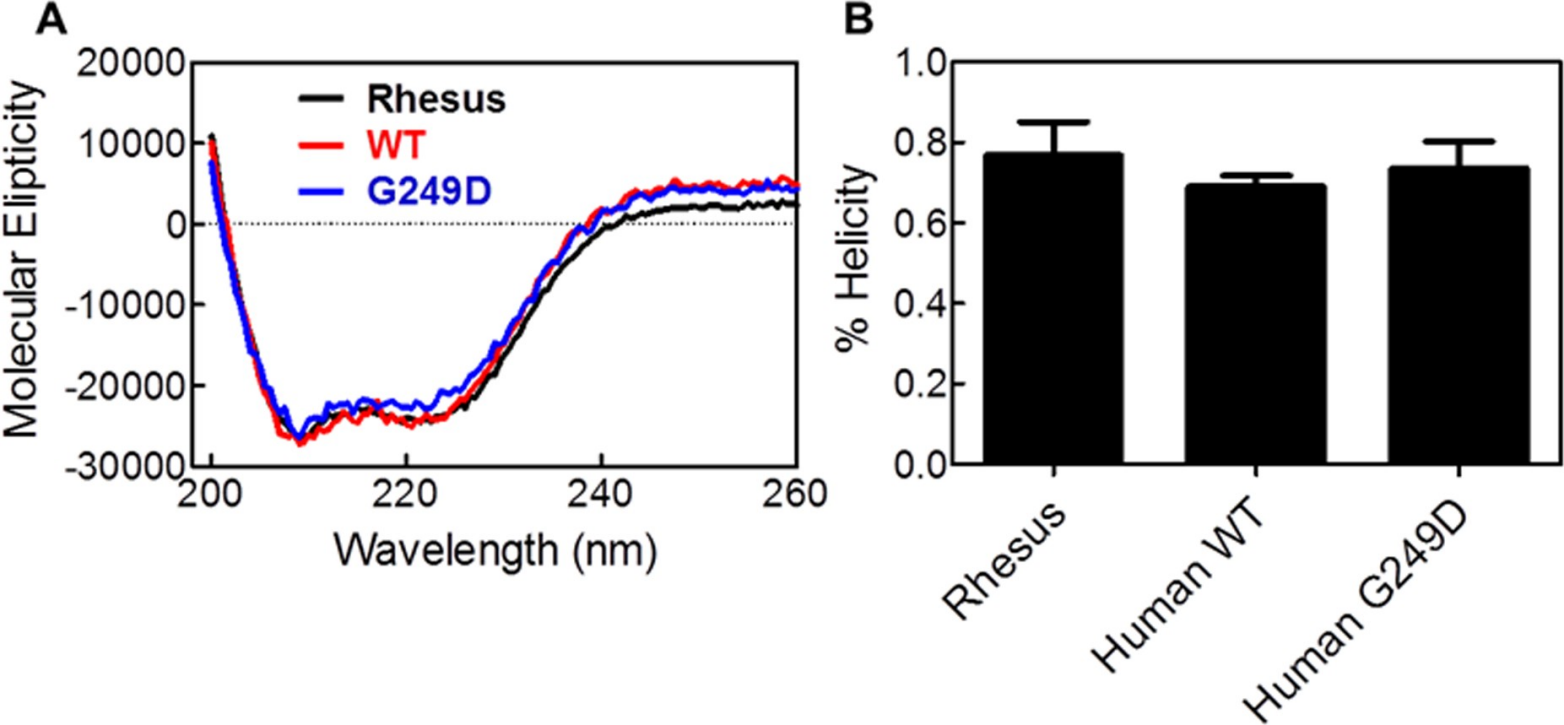
G249D polymorphism in human Trim5*α* does not affect *α*-helical content. **(A)** Circular dichroism of purified CC-L2 peptides of rhesus (in black), human WT (in red) and G249D (in green) TRIM5*α* given in molecular elipticity units. **(B)** Helical content is calculated by using Selcon II software. Error bars represent SEM of 4 biological replicates.

In our previous studies, we observed a correlation between α-helical content in the L2 region of rhTRIM5α and the ability of rhTRIM5α to restrict HIV-1 [10]. Further investigation using sm-FRET measurements revealed that the L2 region undergoes dynamic conformational changes which correlated to changes in alpha helical content in the CC-L2 dimer and restriction capacity of the full length rhTRIM5α protein [42]. We used circular dichroism to determine if the G249D mutation alters the secondary structure of the L2 region of huTRIM5α. We expressed and purified truncated versions of WT and G249D huTRIM5α comprising the CC-L2 dimer. rhCCL2 was used as a standard as it has already been characterized in our previous work. CD data showed characteristics of helical peptides having minima at 208 and 222 nm (Fig 2A). We also analyzed the CD data to obtain the α-helical content for CCL2 dimers and obtained percent helicities (Fig 2B). There were no major differences in the spectral shapes of the human or rhesus peptide, and the calculated helical content did not differ significantly from huCCL2 dimers or the G249D mutant. These results suggest that the G249D mutation does not grossly disrupt the secondary structure of the basic dimeric unit of huTRIM5α.

### Human WT and G249D TRIM5α exhibits similar conformational dynamics by sm-FRET

In our previous studies of rhTRIM5α, we found that conformational changes affecting helical content in L2 region played an important role in the restriction [42]. In this study, we observed that the CC-L2 dimer of rhTRIM5α assumed three distinct conformations which exhibited a 25 (Å) displacement. These conformational changes were found to correlate with the changes in the helical content changes in the L2 region of the CC-L2 dimer. Moreover, a correlation between occupancy of these states and restriction activity of rhTRIM5α was observed and led us to propose a model in which the displacement of the SPRY domain, through conformational changes in L2 region, may facilitate the abortive disassembly of viral capsid.

**Fig 3:**
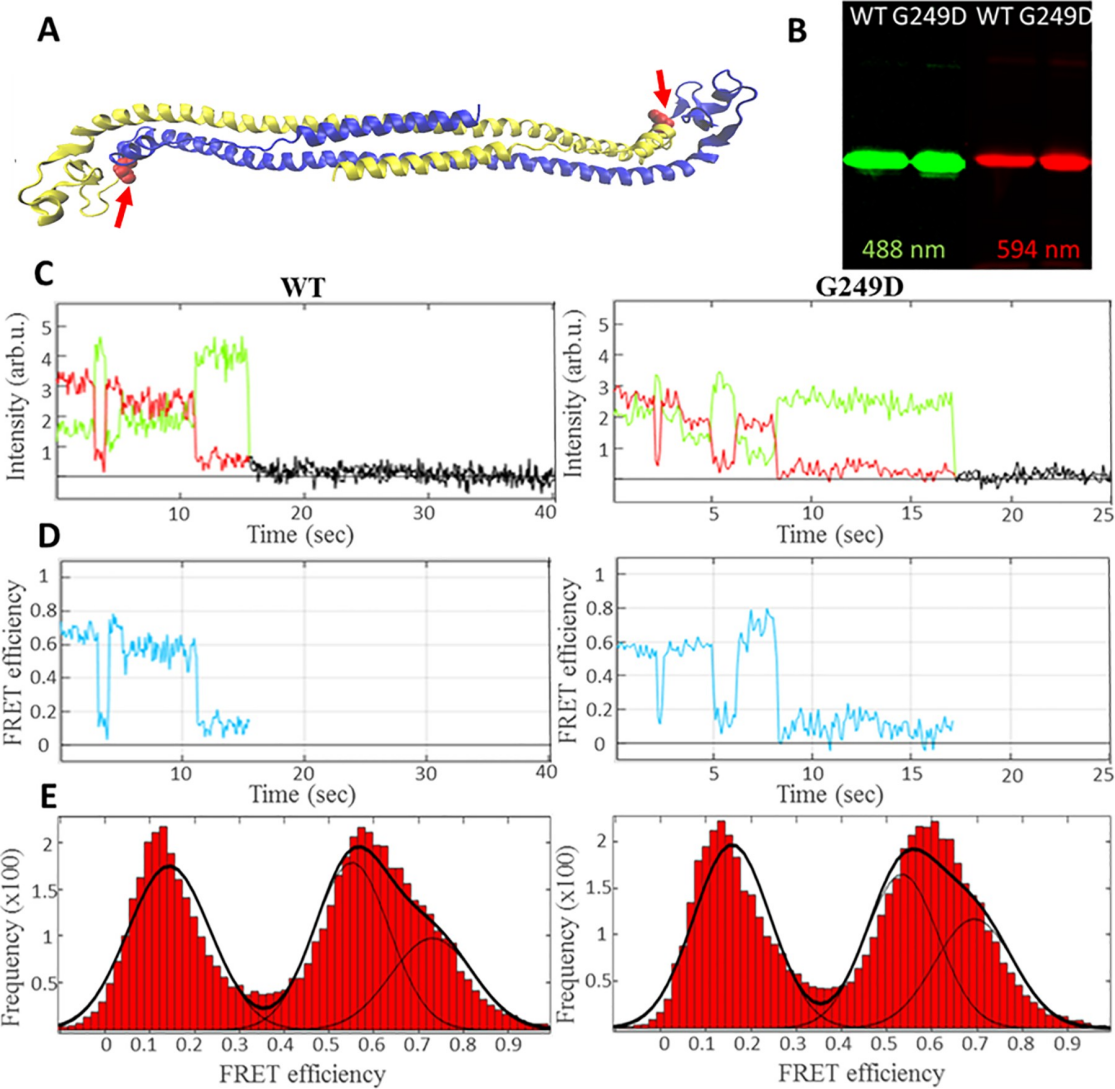
Three different conformational states are observed for human WT and G249D CCL2 dimers. Dually labelled human wt and G249D Trim5α for sm-FRET measurements. **(A)** Cartoon representation of human wt CC-L2 dimers (yellow and blue) depicting location of fluorophore (Alexa 488 by green and Alexa 594 by red pentagon) conjugation and G249 residue (red arrows). **(B)** Gel electrophoresis of purified and dually labelled CC-L2 peptides imaged by using green (526 nm) or red (610 nm) emission filters. **(C)** Representative fluorescence intensity changes over time (donor shown in green, acceptor in red) obtained by TIRF microscopy for WT (left panel) and G249D (right panel) mutant respectively. **(D)** Calculated FRET efficiencies for CCL2 Dimers for WT (left panel) and G249D mutant (right panel) respectively. **(E)** Composite histogram of FRET efficiencies compiled from individual traces and fit to three Gaussian distributions for WT (left panel) and G249D mutant (right panel).

We therefore hypothesized that G249D mutation located in the L2 region may alter the conformational dynamics of the huCCL2 dimer and used sm-FRET to test our hypothesis. We purified CC-L2 dimers of TRIM5α containing a cysteine at the C-terminal and labelled them using maleimide chemistry with donor (Alexa 488) and acceptor (Alexa 594) dyes (Fig 3A). We used TIRF microscopy to collect emissions originating from single chromophore pairs over time and analyzed these data as described previously [42].

Representative traces (intensity versus time) for wild type and G249D CCL2 dimers showed anticorrelated donor and acceptor signal with a single step photobleaching event (Fig 3C). Similar to our previous study of the rhesus CC-L2 dimer, we observed the human CC-L2 dimer undergoes spontaneous transitions between three distinct conformations, with three apparent FRET states. Calculated FRET efficiencies show single step transitions between low and high efficiency states (Fig 3D, WT and G249D respectively). The frequency distributions for different FRET states were obtained (Fig 3E) and fitted with Gaussians centered at around 0.2, 0.6 and 0.8 for WT and G249D respectively representing the distances of 68 (Å), 51 (Å) and 43 (Å) between donor and acceptor chromophores. The lower efficiency state (0.2) has the highest sampling frequency (45% for WT and 43% for G249D) while the least occupied state is the highest efficiency state (0.8, 31% for both WT and G249D). The mid-efficiency state (0.6) was occupied 26% of the time for G249D and 24% of the time for WT. The lack of significant differences between WT and G249D dimers in the FRET state occupancy indicates that G249D substitution does not alter conformational changes in the CC-L2 dimer.

**Fig 4:**
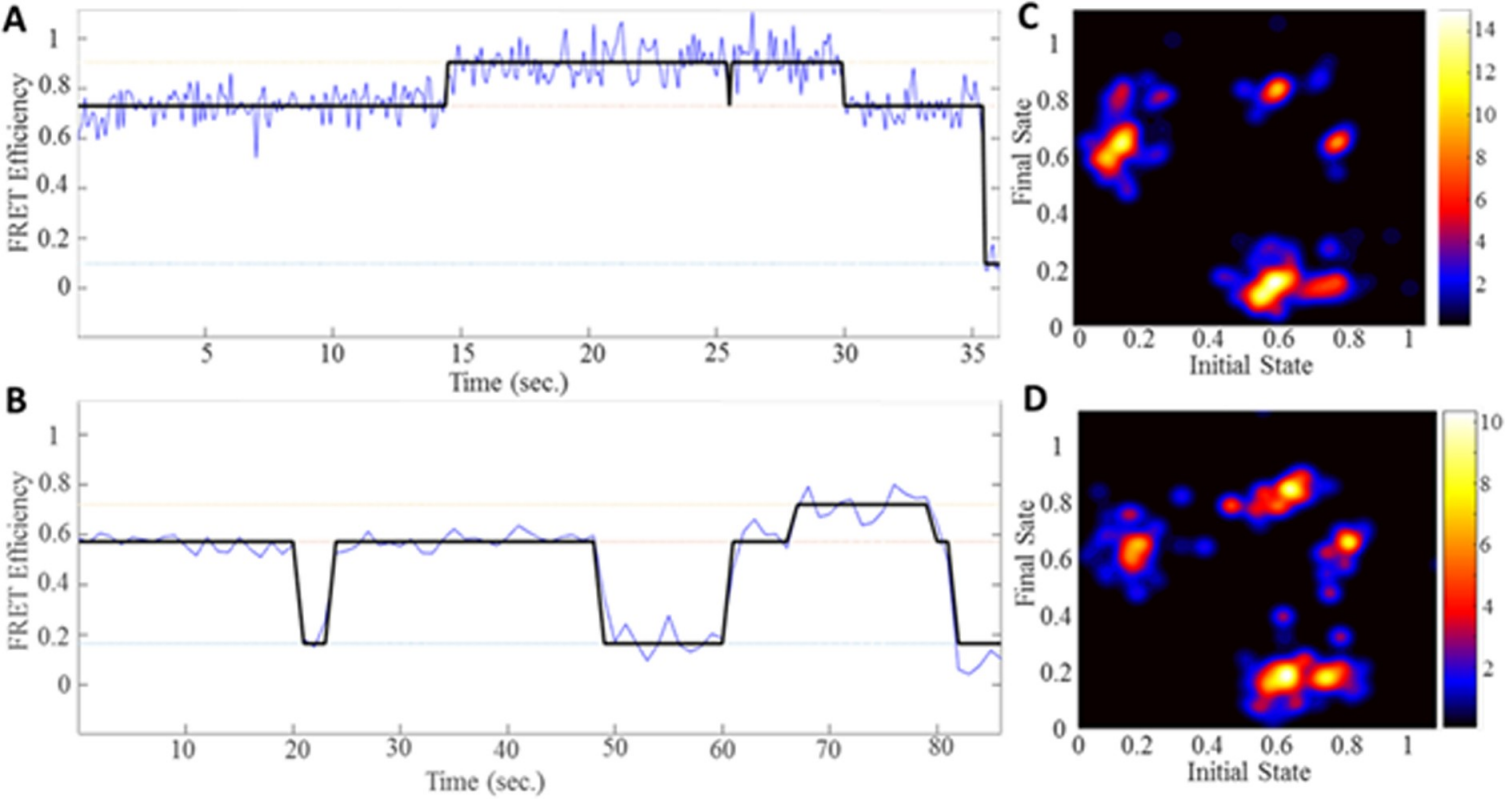
Human WT and G249D TRIM5α exhibits similar conformational dynamics by Hidden Markov Model analysis. Idealized FRET traces were obtained by fitting each trace to a three state Markov Model (in black) for WT **(A)** and G249D **(B)** CCL2 dimer (in blue). Transition density plots were generated from the modelled FRET trajectories for WT **(C)** and G249D **(D)** CCL2 dimer (68 and 52 traces with 240 and 185 transitions were used for generation of TDP plots for WT and G249D respectively).

To further analyze broadened FRET distributions, we used Hidden Markov Model (HMM). We obtained transitions between noiseless FRET values called idealized transitions. Representative idealized transitions for WT and G249D dimers involving three different FRET efficiency states are given in Fig 4A and 4B. Cumulative frequency distributions (Fig 3E) are in good agreement with our analysis using HMM. We compared the connectivity of the individual FRET states using these idealized transitions. Transitions between 0.2 and 0.6 efficiency states (55% of the transitions) were more likely than transitions involving highest efficiency state 0.8. The least common transitions were between 0.6 and 0.8 which was also observed with rhesus CCL2 dimers (%15 of the transitions). Transition density plots showing the connectivity of individual states were also obtained using the idealized FRET transitions. They also reveal no significant difference between WT and G249D (Fig 4C and 4D). These data demonstrate that conformational dynamics of the human CC-L2 dimeric units of TRIM5α are not affected by the G249D polymorphism.

### Molecular dynamics simulations reveal differences in BBox2 positioning of Human WT and G249D TRIM5α

Given that biochemical and biophysical examination of the human CC-L2 dimer did not reveal any apparent defect in secondary structure or conformational dynamics, we next considered how the G249D mutation may influence other aspects of TRIM5α function. Given the location of G249 in the hairpin region of the dimer (Fig 3A), we reasoned that the G249D mutation may influence the function of the adjacent BBox2 domain to mediate the higher order assembly of TRIM5α dimers [5, 58, 59]. To test this hypothesis, we used molecular dynamics simulations to determine how the G249D mutation influences the orientation of the BBox2 domain in the TRIM5α dimer and the stability of inter-dimeric BBox2 interfaces which support the assembly of TRIM5α assemblies [9, 15, 16].

**Fig 5:**
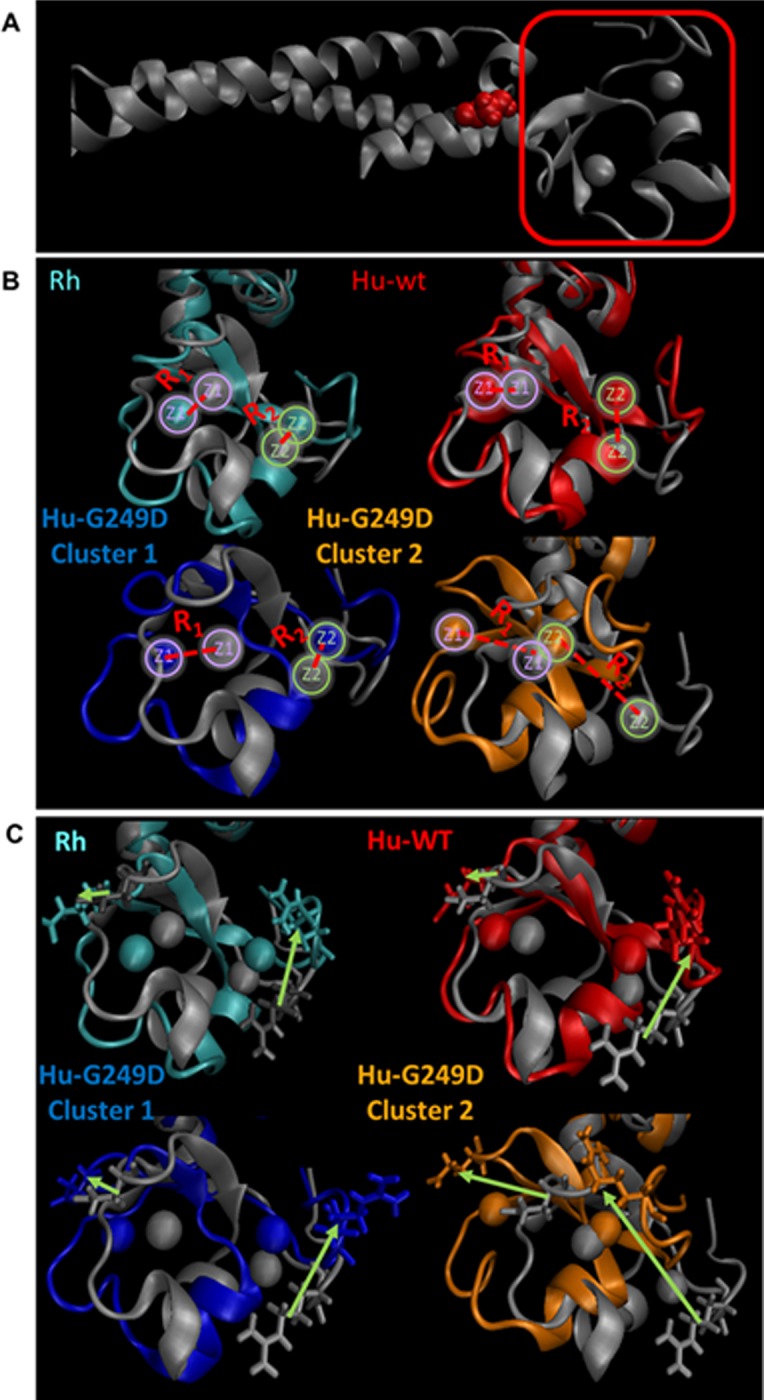
Human G249D CCL2 dimer has distorted placement of BBox-2 domain compared crystal structure. **(A)** Crystal structure of rhesus CCL2 given in silver. Trajectories from MD simulations classified into clusters according to their RMSD (threshold value of 2.25 Å). **(B)** Rhesus CCL2 dimer major cluster (in cyan, 64%), human WT CCL2 dimer major cluster (in red, 89%), human G249D CCL2 dimer cluster 1 (in blue, 64%), human G249D CCL2 dimer cluster 2 (in orange, 10%). R_1_ and R_2_ represents the distances between zinc ions and zinc ions are color coded (purple for the first and green for the second zinc ion). **(C)** Representative side chains are depicted for each cluster. Arrows represent how the side chains displaced compared to rhesus crystal structure.

In order to estimate effect of G249D mutation on structure of individual dimers, we performed molecular dynamics simulations of rhesus WT, human WT and G249D monomers, using the published structure of the rhTRIM5α BBox2-CC-L2 dimer as an initial reference structure [15]. In Fig 5A, the crystal structure of rhTRIM5α used for homology model is shown and the region of interest is depicted. Trajectories, which are snapshots of the simulated structures from 100 ns simulation runs with 1ps time steps (10^5^ trajectories), were analyzed by clustering structures based on root mean square deviation (RMSD) between them. We aligned coil-coil regions of the structures and added a structure to a cluster when its RMSD value is less than 2.25 Å. Rhesus WT and human WT show only one main cluster of structures with populations of 64% and 89% respectively (Fig 5B). In contrast G249D exhibits a significantly populated second cluster (cluster 2, 10%) in addition to the main cluster (cluster 1, 64%) (Fig 5B). In Fig 5, our results are shown in comparison to crystal structure of the rhesus dimer (silver). We used the crystal structure of rhTRIM5α as the reference structure and examined deviations from it. In our production runs the BBox2 domain moves away from the original rhesus crystal structure which can potentially affect the interface between neighboring dimers. In order to quantify these deviations in BBox2 domain, we monitored the movement of zinc ions, as coordination of zinc ions by BBox2 residues is critical to its biological function [9, 60, 61]. As shown in Fig 5B, we measured the distances between zinc ions (red lines, zinc ions are color coded with green and purple) given in Table 1. Our simulations revealed a minor deviation from crystal structure for the rhesus monomer (3.3 Å, Table 1) and zinc ions move together with the coordinating residues (Fig 5C). This deviation significantly increases for human proteins, particularly G249D mutant in cluster 2 (10 Å). Structures in this cluster are likely to be deformed by interaction of residue 249D with residue Arg97 on the loop where residue Glu100 located (E102 residue for rhesus which was shown to be important in oligomeric interactions) [15]. In order to quantify the deviation of BBox2 domain as whole, we calculated RMSD values for each cluster by aligning the coiled coil domain (Table 1). The highest RMSD was observed for the G249D cluster 2, which represents the most distorted structures. Our MD simulation results suggest that G249D substitution alters the positioning of BBox2 domain and distorts three-fold symmetry of the trimers which is necessary for hexagonal lattice formation and/or stability.

**Table 1:** Root mean square values for each cluster.

	R_1_ (Å)	R_2_ (Å)	R_avg_ (Å)	RMSD (Å)
Rh WT (64%)	3.35	3.36	3.35	2.34
Hu WT (89%)	6.42	2.92	4.67	3.15
Hu G249D (64%)	5.37	5.98	5.67	2.84
Hu G249D (10%)	10.4	9.26	9.98	4.48

**Fig 6:**
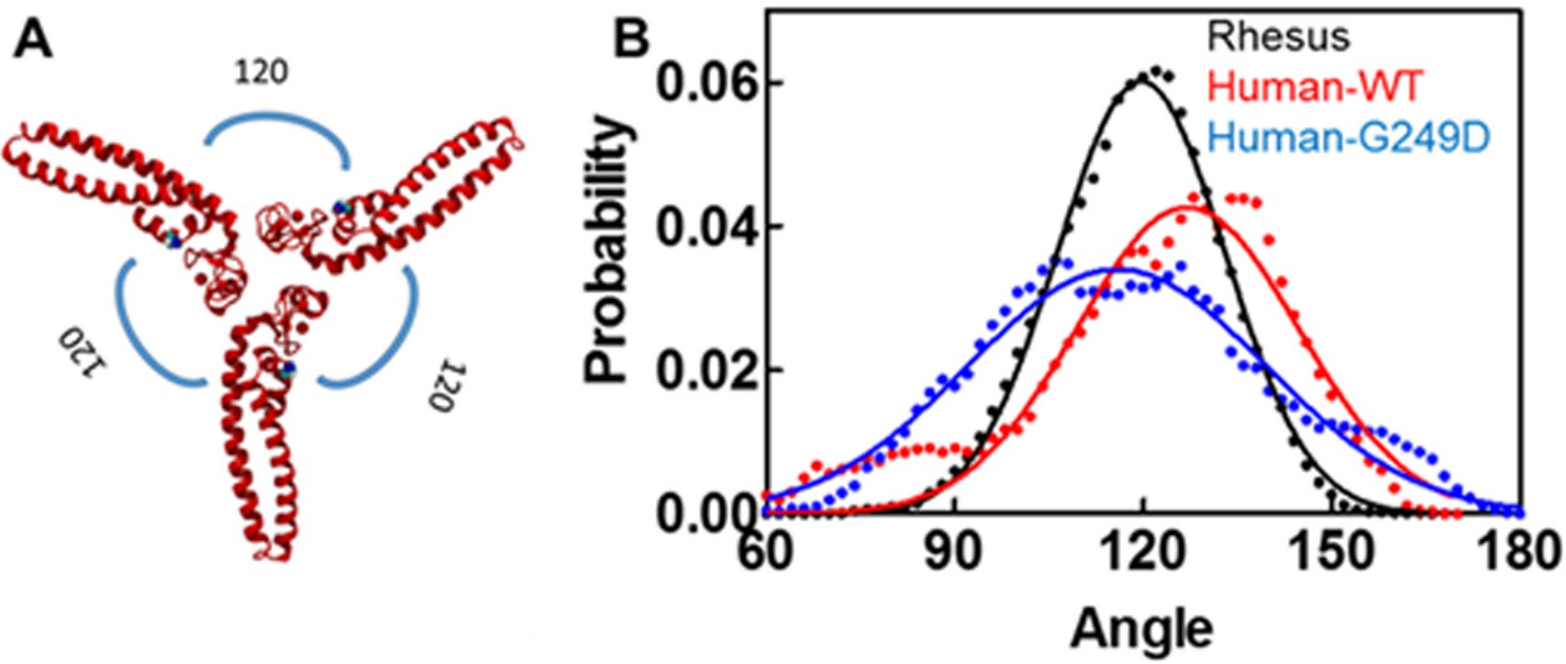
Human WT and G249D Bcc miniTRIM oligomers exhibit wider angle distributions than rhesus miniTRIM. **(A)** Rhesus Bcc miniTRIM structure with representative angles. **(B)** Probability distribution of angles for rhesus (in black), human WT (in red) and G249D (in blue) Bcc miniTRIMs obtained from MD simulations of our homology models (circles) and Gaussian fits (solid lines). The angles are defined by the angle between Cα atoms of residues 117 and 174 for rhesus and 115 and 172 for human oligomers and gated for 2 degrees to obtain frequency distribution.

**Fig 7:**
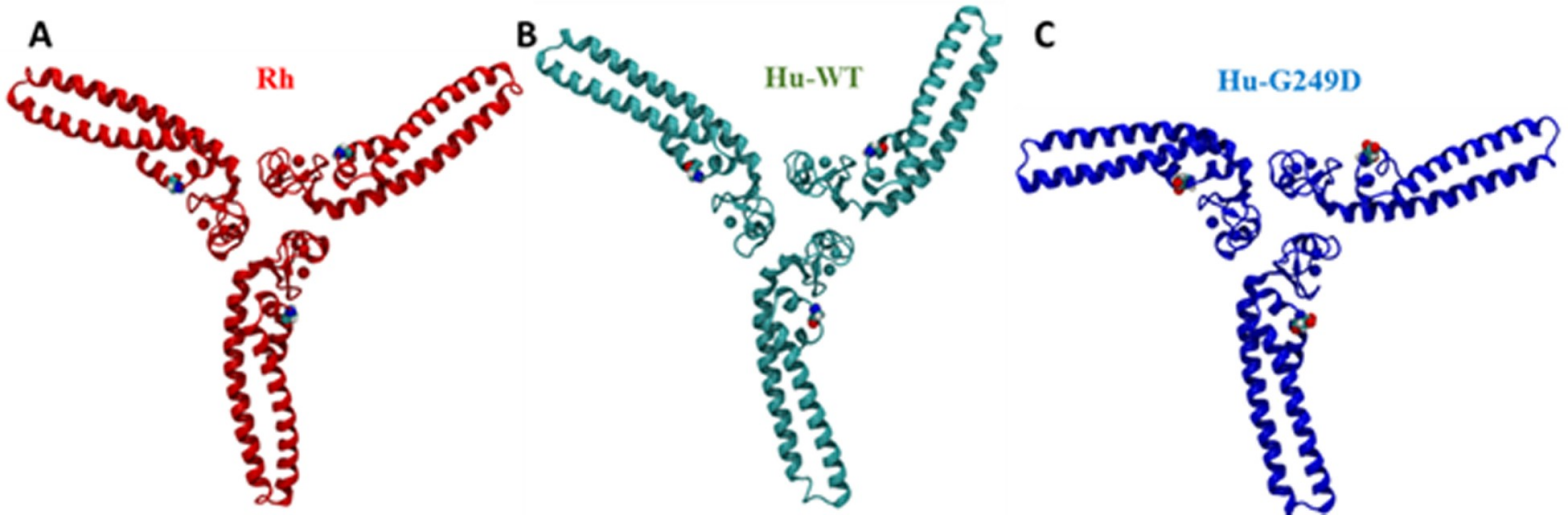
Representative structures of Human WT and G249D Bcc miniTRIM oligomers by MD simulations showing distortions in G249D miniTRIM. We used Bcc miniTRIM structures starting geometries for our homology models and obtained MD simulations with trajectories showing distortions in three-fold symmetry. **(A)** Rhesus Bcc miniTRIM (in red) **(B)** Human WT Bcc miniTRIM (in cyan) **(C)** Human G249D Bcc miniTRIM (in blue).

We also used molecular dynamics simulation techniques to study structural stability of higher order assemblies of rhesus, human WT and G249D TRIM5α. For our studies, we used Bcc miniTRIM structures as starting geometries for our simulations because they recapitulate the positioning of the BBox2 in the context of the TRIM5α dimer and can be used to study higher order hexagonal assemblies of TRIM5α [15]. We ran our simulations for miniTRIM oligomers for 100 ns under constant pressure and temperature conditions with 1ps time steps. To understand the differences between rhesus, human WT and G249D miniTRIMs (or trimers), we measured the angle between adjacent monomers (as shown in Fig 6A) for each trajectory and obtained probability distributions by gating the data for 2 degrees. Angle was defined as an angle between vectors defined by Cα atoms of residues 117 and 174 for rhesus and 115 and 172 for human. In the ideal case of a planar structure, all angles are expected to be 120 degrees. Fig 6B shows the probability distributions of such angles along with Gaussian fits of probability distributions. Rhesus WT miniTRIM exhibited a narrower distribution around 120 degrees compared to both human WT and G249D. In both human cases, distributions of angles are wider, with tails reaching to extreme angles of 60 and 180 degrees. Moreover, the center of the Gaussian deviates from 120 degrees, indicating deviation from the idealized three-fold symmetry. While human trimers exhibited wider angle distributions than rhesus trimers, G249D substitution resulted in the lowest probability at the center angle of the Gaussian distribution. These significant variations from the center represents the distortions in three-fold symmetry axis of the BBox2/CC head of TRIM5α. Fig 7 shows examples of such distorted structures (C) in comparison to perfectly symmetric crystal structure of rhesus WT (A). As can be seen from these examples, human trimers can deviate from three-fold symmetry axis significantly causing decreased stability of the hexagonal lattice and/or defects in formation of such lattices. These effects are more pronounced for the G249D trimer. These results suggest that the G249D polymorphism may impact the orientation of the BBox2 prior to higher order assembly as well as influence the structural features of BBox2 assemblies.

### Cytoplasmic body formation is reduced by G249D polymorphism in human TRIM 5α

Our modelling results indicated that the G249D polymorphism can alter the formation of higher assemblies of huTRIM5α. In order to test that, we quantified formation of cytoplasmic bodies in CRFK cells stably expressing YFP tagged huTRIM5α (Fig 1). The ability to form cytoplasmic bodies in cells reflects the ability of TRIM5α to form higher-order assemblies, and it has been known that there is a direct positive correlation between cytoplasmic body formation and restriction activity [10, 26, 62].

**Fig 8:**
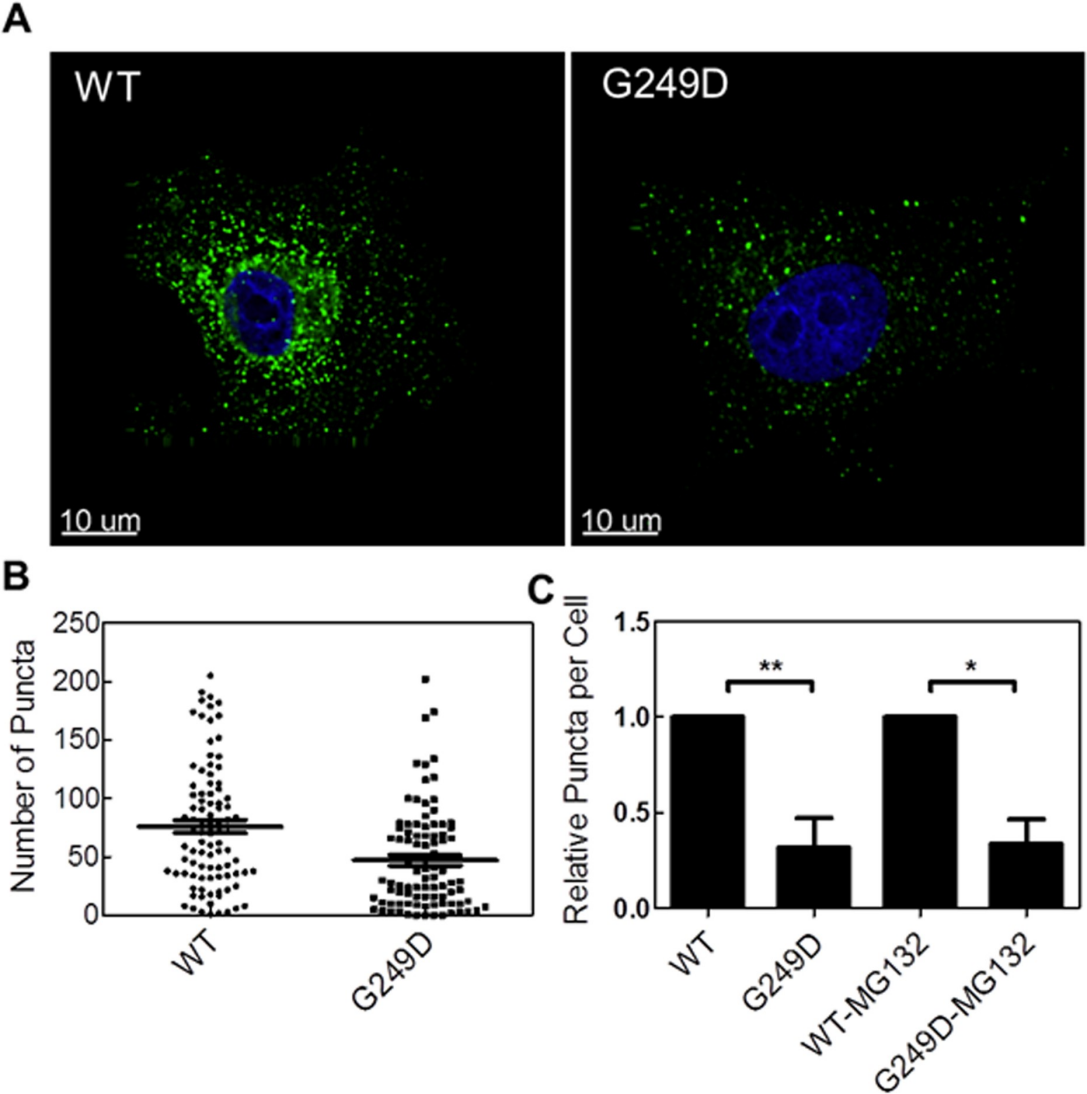
Cytoplasmic body formation is reduced by G249D polymorphism in human Trim5α. **(A)** Representative cell images for CRFK cells stably expressing WT or G249D mutant Trim5*α* (green) with DAPI stain (blue). **(B)** Number of cytoplasmic bodies per cell for CRFK stably expression YFP tagged WT or G249D mutant Trim5*α* were determined from surface analysis (C) Relative puncta is computed by normalization to average number of cytoplasmic bodies per cell for cells expressing WT Trim5*α* and computed for five biological replicates. Error bars represent SEM for five biological replicates (p<0.05 compared to the WT for no treatment and treatment cases).

Fluorescent microscopy revealed that both proteins form cytoplasmic bodies in CRFK cells (Fig 8A). Using algorithm-based detection of cytoplasmic bodies, we quantified number of cytoplasmic bodies per cell, which revealed that the average number of puncta per cell line is (Fig 8B) decreased with G249D substitution. To quantify the decrease in cytoplasmic bodies, we normalized average number of cytoplasmic body per cell to the WT YFP-huTRIM5α expressing cells for five biological replicates (Fig 8C). We obtained a significant decrease in cytoplasmic bodies with G249D mutation compared to WT. The decreased cytoplasmic bodies by the G249D mutant indicates a defect in higher-order assembly, consistent with the results of our MD simulations.

## Discussion

Although the magnitude of HIV-1 restriction by huTRIM5α in single cycle infectivity assays is modest, it is worth noting that numerous studies have found that TRIM5α proteins which exhibit modest restriction in single cycle infectivity assays can facilitate immunity *in vivo* by involving components of the immune system. Moreover, TRIM5α was identified as one of the host factors mediating interferon inhibition of HIV-1 in an unbiased CRISPR screen along with MxB, IFITM1, and Tetherin/BST2[63]. For example, primary cells from rhesus macaques inhibit HIV-1 infection significantly, but measurable infection is observed, even at low doses of virus [64–66], yet TRIM5α is known to provide sterilizing immunity against HIV-1 in rhesus macaques. Similarly, Richardson et al. have observed that mCherry-huTRIM5α provides sterilizing immunity against HIV-1 infection in a humanized mouse model of HIV-1 infection despite observing restriction in vitro that was incomplete and less potent than rhTRIM5alpha [67]. This enhanced potency *in vivo* is likely due to a combination of the ability of TRIM5α to generate K63-linked ubiquitin chains which upregulate the expression of other antiviral genes [68] and the cumulative effect of modest restriction amplified over many replication cycles. Similarly, the observation that the G249D mutation induces a reproducible but modest relief of HIV-1 restriction by huTRIM5α in our experiments correlates to a significant increase in higher susceptibility to HIV-1 infection and higher viral titers in HIV infected patients [39–41].

In this study, we investigated the underlying mechanism behind the reduced restriction of HIV- 1 by G249D huTRIM5α compared to WT. We investigated two different aspects that can be affected by G249D substitution: secondary structure related changes including conformational dynamics and formation of higher order assemblies that are necessary for restriction activity.

The secondary structure of the TRIM5α dimer is known to be an important factor in restriction activity [10, 12, 14]. As the G249D substitution is located in L2 domain, we hypothesized that it was causing alterations in helical content. We obtained helical content for both WT and G249D by using CD spectroscopy which revealed that the G249D dimer exhibits secondary structure similar to WT huCCL2 dimer. The conformational changes in L2 region is suggested to be playing a role in restriction activity of TRIM5α [42]. However, sm-FRET data also depicted similarity between G249D and WT CCL2 dimers. Although we observed that the mutations in the L2 region can be critical for secondary structures and dynamic conformational changes in our previous studies, G249D substitution does not affect the protein structure in a direct manner.

To further investigate effects of G249D substitution on protein structure, we used homology modelling. Our homology models revealed that the G249D mutation was present on the hairpin of the antiparallel dimer, in a position that may be critical for interactions with adjacent BBox2 domain. A hydrophobic patch on BBox2 domain surface was identified as essential for maintaining inter-dimeric contacts required for the formation of TRIM5 assemblies in literature[9]. Disruption of the hydrophobic interactions among adjacent hydrophobic BBox2 patches resulted in lower binding affinity of TRIM5α to HIV-1 capsid and lower restriction activity. For this reason, we wanted to investigate impact of G249D substitution on three-fold symmetry of higher order assemblies which is mediated by BBox2 domain. For our computational studies, we used miniTRIM structures as surrogates for BBox2/CC core of full TRIM5α [15]. Our simulations depicted large distortion from three-fold symmetry by human trimers. This difference in behavior of rhTRIMα and huTRIMα is consistent with previous observations that huTRIMα can form elongated assemblies in cells while rhTRIM5α assemblies are predominately spherical, as observed by fluorescent microscopy [26]. We also noted that the probability distribution of such angles between adjacent monomer were widest for G249D. We also ran RMSD analysis on individual rhesus human WT and G249D miniTRIM dimers and observed the largest distortions from the rhesus crystal structure by the G249D mutant, implying that this substitution can affect formation and/or stability of hexagonal assemblies.

Restriction of N-MLV is not affected by G249D polymorphism. It has been shown that human TRIM5α provides potent block against N tropic viral capsid [69] and this restriction is dependent on capsid recognition by SPRY domain [70]. In the presence of high affinity capsid binding the defects in assembly or BBox2 domain interactions can be compensated by high affinity binding to N tropic capsid. This result is also consistent with previous studies which observed the BBox2 domain is dispensable in the case of TRIM-Cyp, which has a higher affinity for HIV-1 capsid, while required for restriction of HIV-1 by rhTRIM5α [71]. In this case, the G249D polymorphism impacted the ability of huTRIM5α to enhance low affinity binding to HIV-1 capsid but was dispensable in restriction of N-MLV restriction, presumably due to the higher affinity of the huTRIM5α SPRY domain for N-MLV capsid.

Cytoplasmic bodies formed in cells expressing TRIM5α are suggested to be manifestations of higher order hexagonal assemblies [14, 26], such as those observed *in vitro* by Ganser-Pornillos *et al* [18, 20]. Our computational studies implied that hexagonal lattice formations are altered by the G249D substitution, which we confirmed by examining cells expressing YFP-tagged forms of these full-length proteins. These cell lines expressing comparable amounts of TRIM5α formed cytoplasmic bodies manifested as puncta in fluorescence images. G249D protein formed fewer assemblies than WT protein, in agreement with our predictions G249D substitution decreases tendency for formation of three-fold symmetric assemblies. We have shown that reduced antiviral activity of G249D huTRIM5α is caused by the defects in assembly.

## Conclusions

In this study, we investigated a single nucleotide polymorphism in huTRIM5α, which is associated with increased HIV-1 susceptibility and rapid disease progression. We examined the mechanism behind the reduced antiviral activity by variety of biophysical methods and eliminated other possibilities related to secondary structure and conformational dynamics except higher-order assembly formation. Molecular dynamic simulations and in vivo imaging studies revealed that defects in assembly reduce antiviral activity of G249D polymorphism in huTRIM5α.
